# Supplementary material for: Crystal nuclei templated nanostructured membranes prepared by solvent crystallization and polymer migration
Source: Nat Commun. 2016 Sep 19;7:12804. doi: 10.1038/ncomms12804 (PMC5031797; doi:10.1038/ncomms12804)
Supplement: Supplementary Information — Supplementary Figures 1-20, Supplementary Tables 1-4, Supplementary Notes 1-5 and Supplementary References [file ncomms12804-s1.pdf]

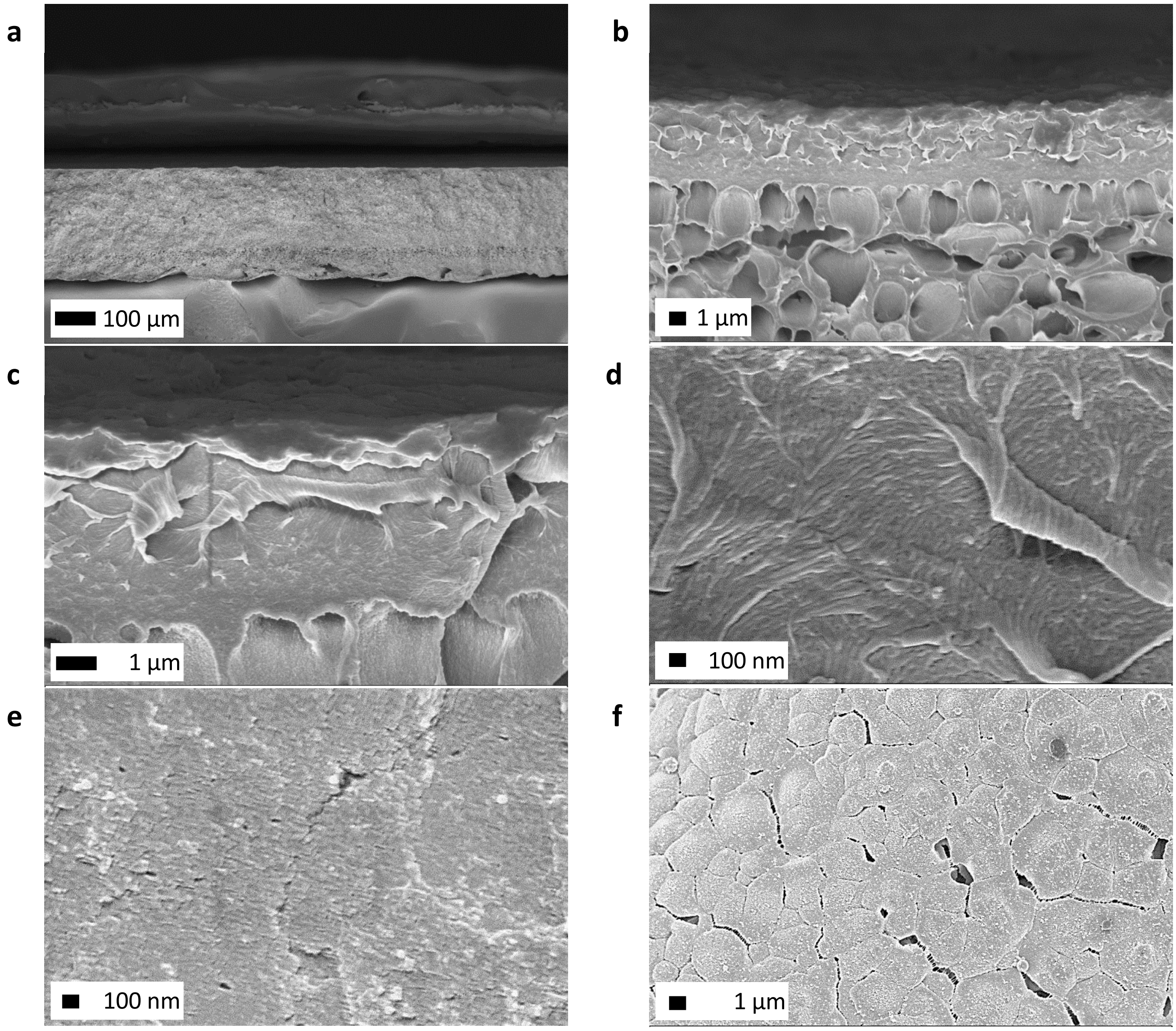


**Supplementary Figure 1. SEM images of the CCD Al/Al NMP PVDF membrane.** (**a**) Cross-sectional overview; (**b,c**) close view showing the separation layer; (**d**) high-magnification image at the separation layer; (**e**) surface of the separation layer; (**f**) surface of the back side.

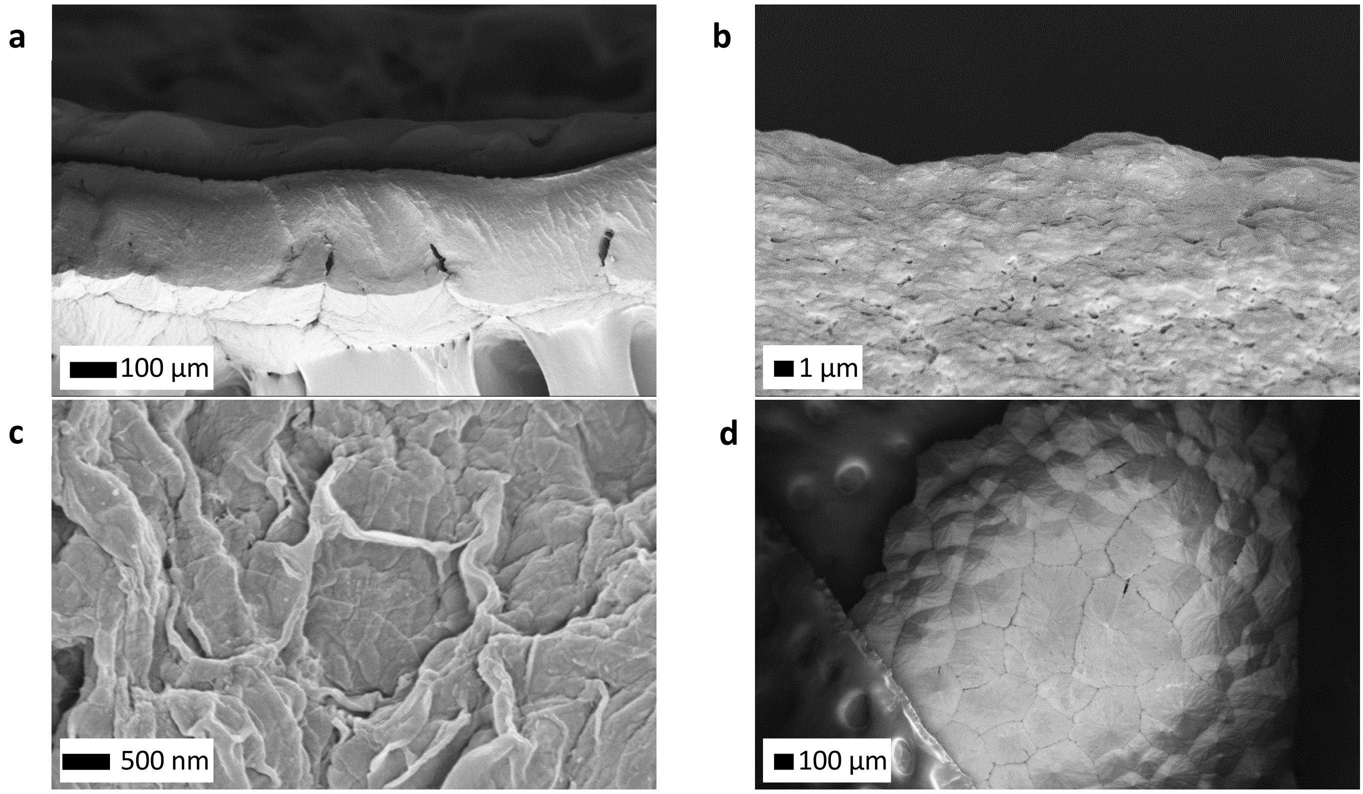


**Supplementary Figure 2. SEM images of the CCD Al/Al DMAc PVDF membrane.** (**a**) Cross-sectional overview; (**b**) close view showing the separation layer; (**c**) surface of the separation layer; (**d**) surface of the back side

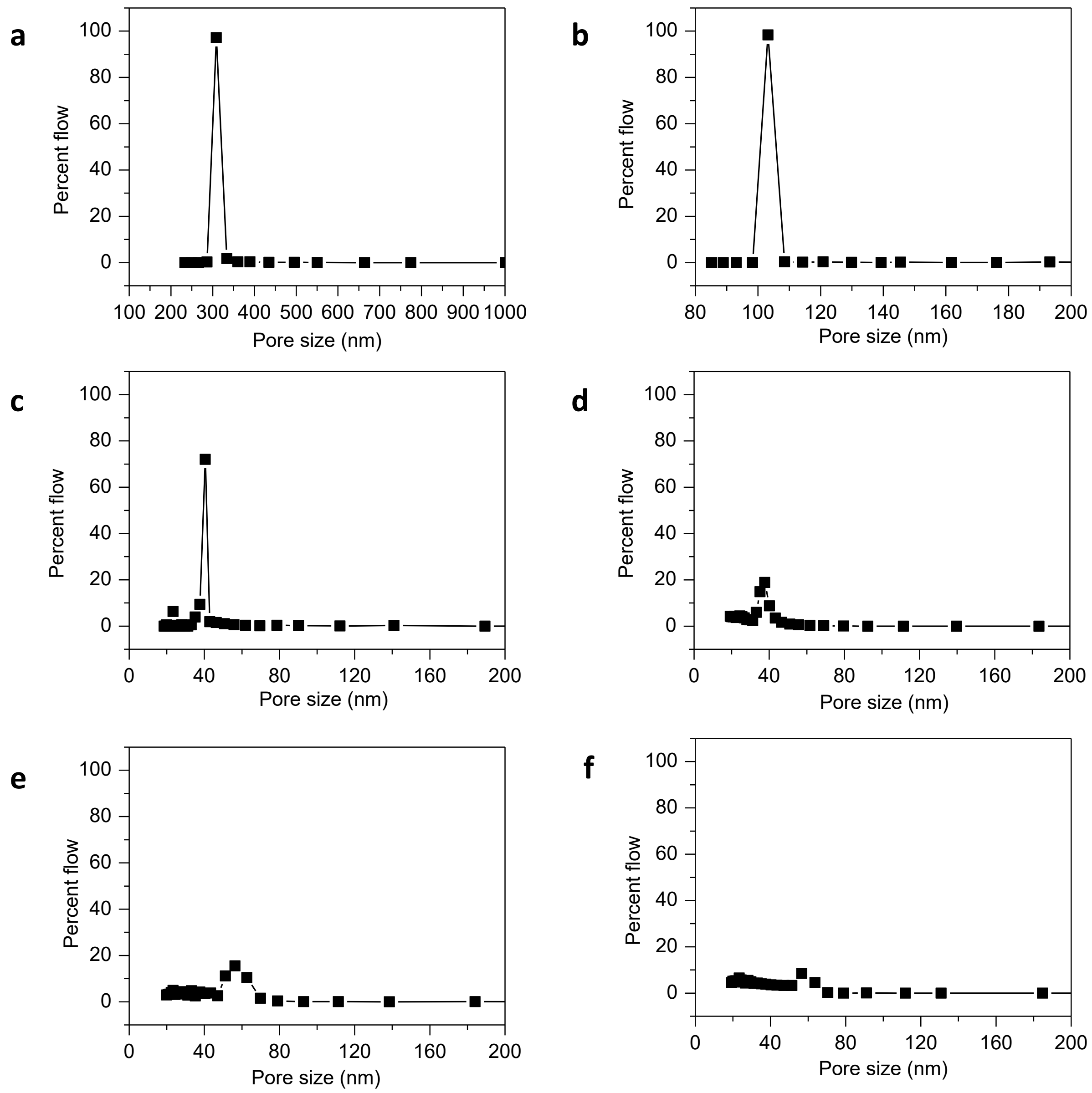


**Supplementary Figure 3. Typical pore size distribution of PVDF membranes measured by the gas-liquid displacement method.** (**a**) CCD Glass/Glass 1 mm sample, which shows a pore size range from 286 to 389 nm and a mean flow pore size of 321 nm; (**b**) CCD Glass/Al 1 mm sample, which shows a pore size range from 103 to 240 nm and a mean flow pore size of 106 nm; (**c**) CCD Al/Al 1 mm sample, which shows a pore size range from 33 to 90 nm and a mean flow pore size of 41 nm; (**d**) NIPS DMSO 1 mm sample, which shows a pore size range from 19 to 80 nm and a mean flow pore size of 37 nm; (**e**) NIPS DMSO 0.3 mm sample, which shows a pore size range from 20 to 79 nm and a mean flow pore size of 57 nm; (**f**) NIPS NMP 0.3 mm sample, which shows a pore size range from 19 to 70 nm and a mean flow pore size of 41 nm.

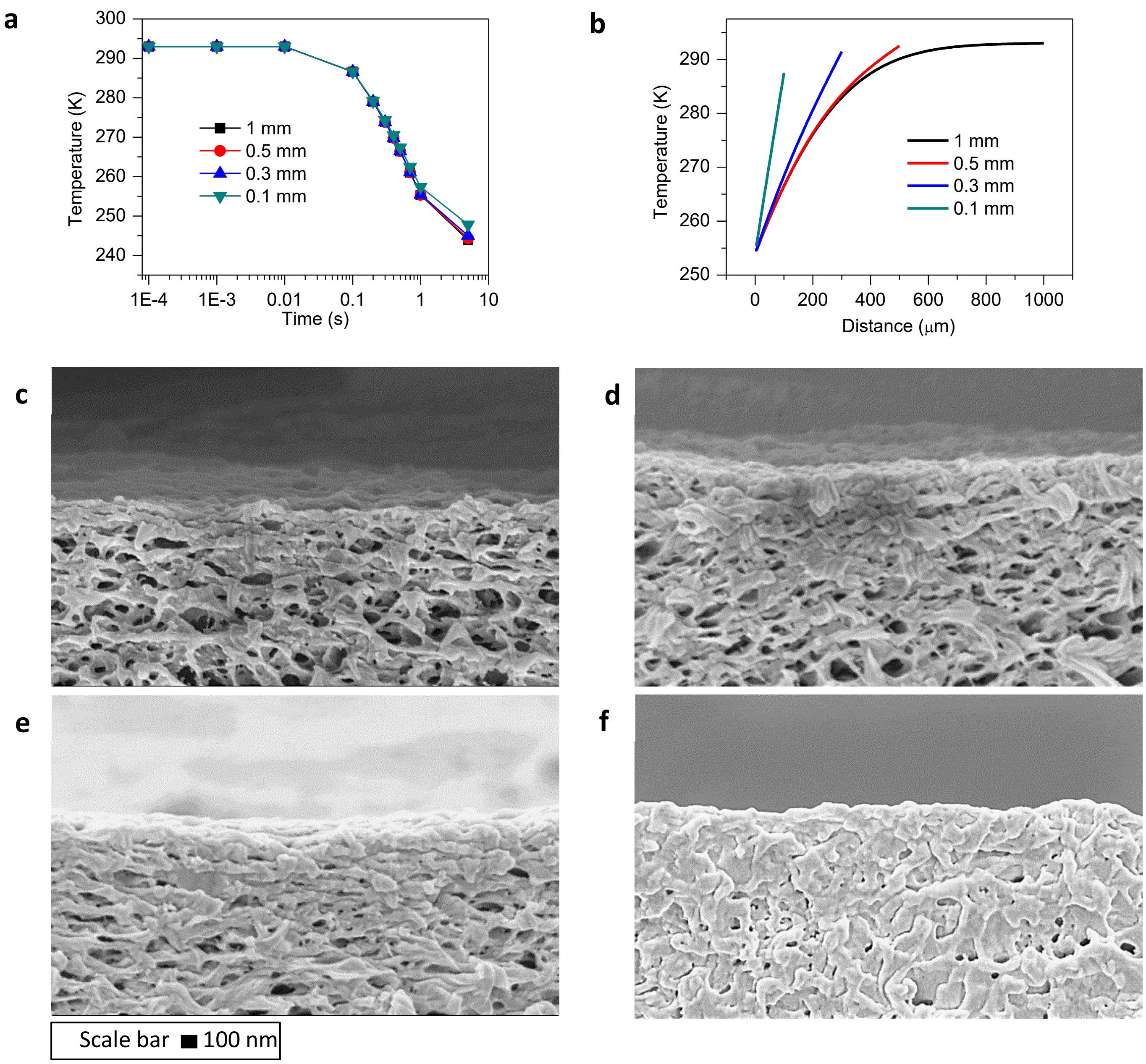


**Supplementary Figure 4. CCD Al/Al PVDF membranes prepared with different casting thicknesses.** (**a**) Temperature change in the polymer film at the position 10 μm away from the cooling interface. (**b**) Temperature profile of the polymer film from the cold end after cooling for 1 s. (**c-f**) Cross-sectional SEM image of the separation layer of the 1mm, 0.5mm, 0.3 mm and 0.1 mm sample, respectively.

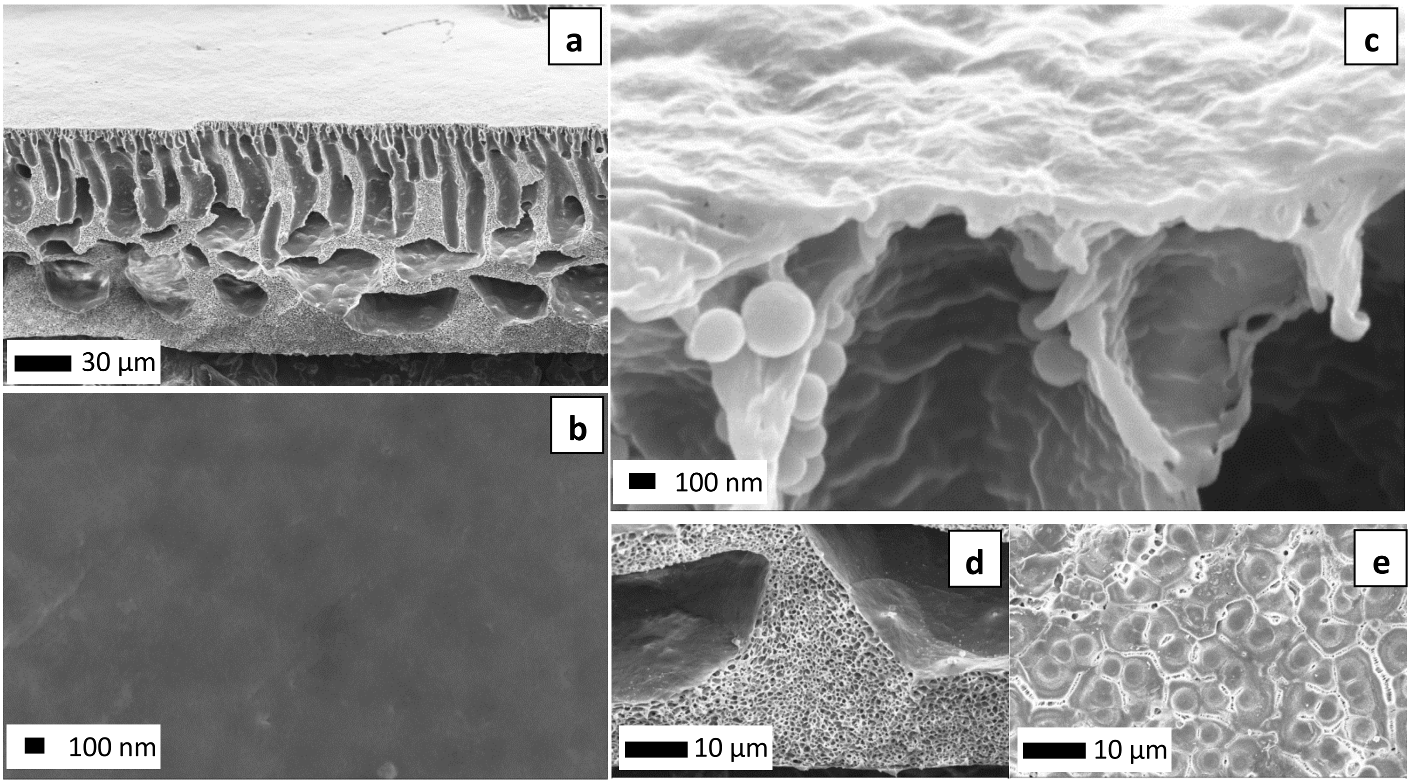


**Supplementary Figure 5. SEM images of the NIPS DMSO PVDF membrane prepared with a 0.3 mm casting thickness.** (**a**) cross-sectional overview; (**b**) top surface; (**c**) close cross-sectional view of the top layer; (**d**) close cross-sectional view of the back side and (**e**) the back surface.

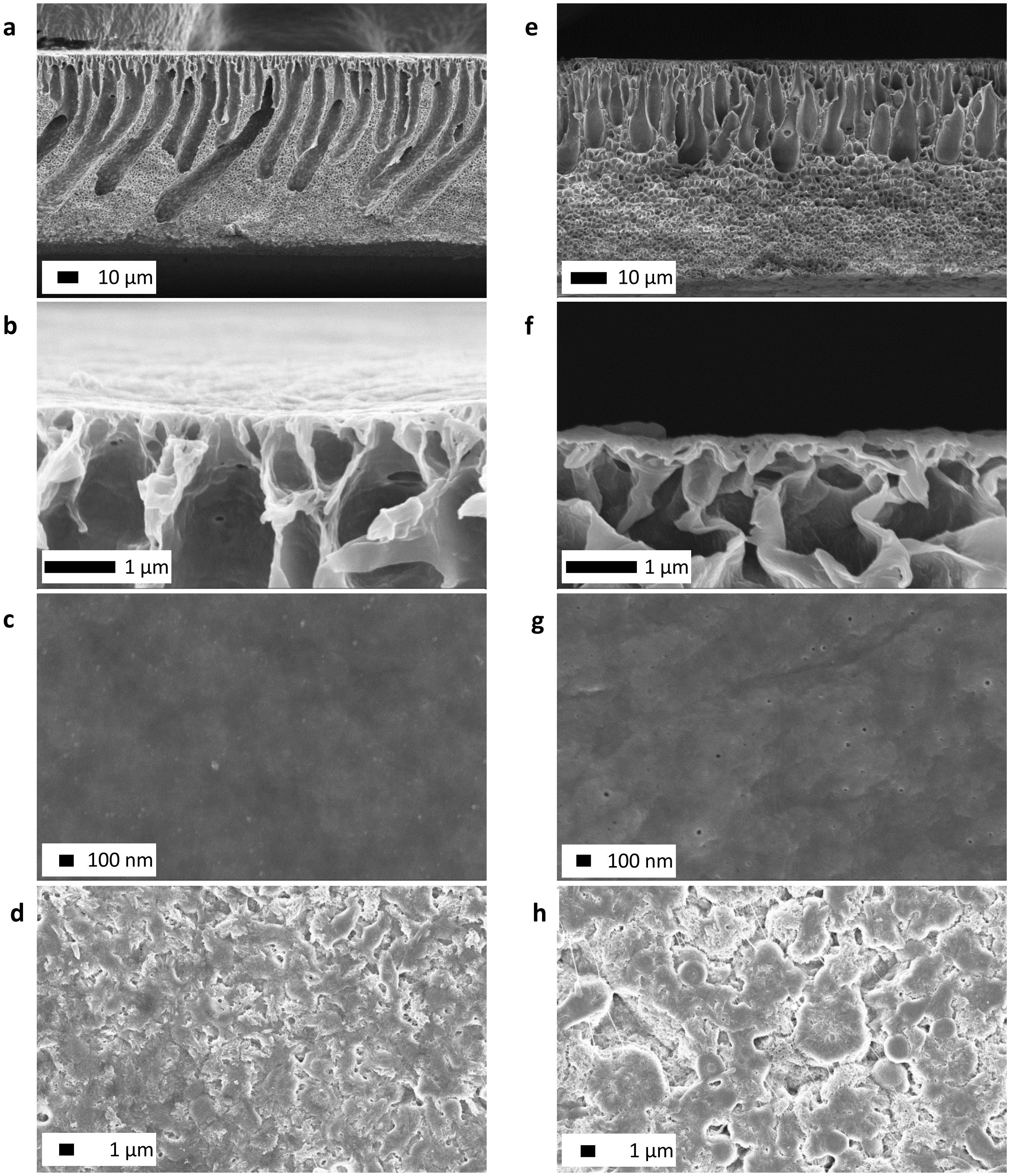


**Supplementary Figure 6.** SEM images of the NIPS NMP (**a-d**) and NIPS DMAc (**e-h**) PVDF membranes prepared with a 0.3 mm casting thickness. (**a, e**) cross-sectional overview; (**b, f**) close cross-sectional view of the top layer; (**c, g**) top surface; (**d, h**) the back surface.

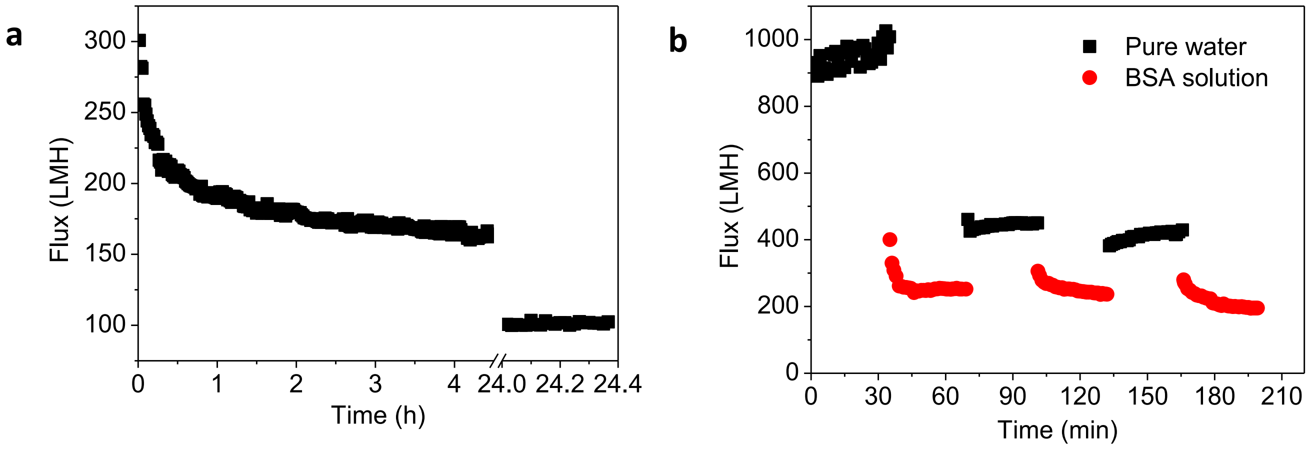


**Supplementary Figure 7. BSA fouling test result of a CCD Al/Al 1.0 mm PVDF membrane.** (**a**) permeation flux v.s. time over 24 hours, (**b**) permeation flux during three cycles of pure water/BSA solution permeation tests. The operating pressure difference was 1 bar, and the membrane was cleaned by ultrasonication after each BSA solution test.

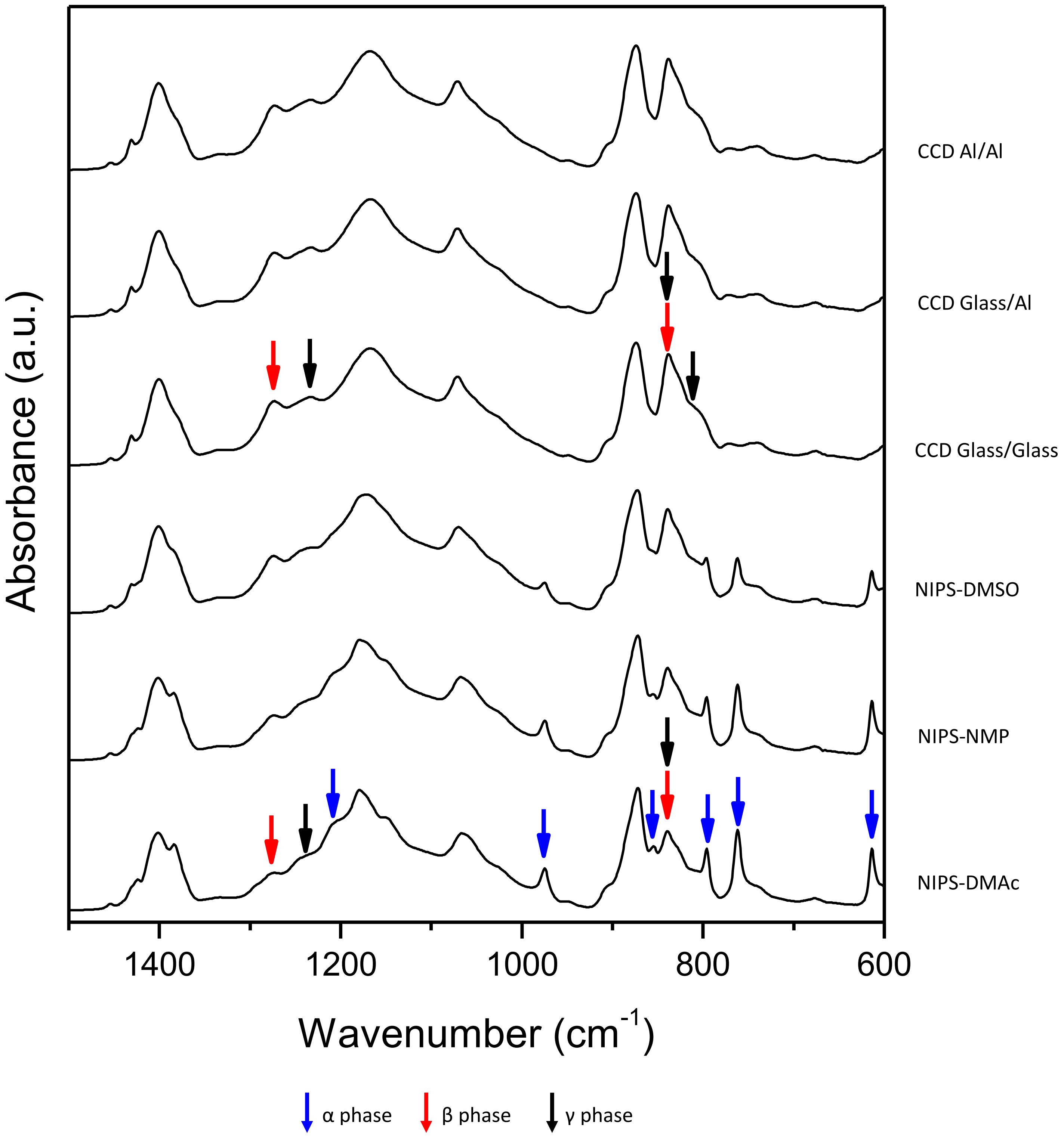


**Supplementary Figure 8. FT-IR spectra of the CCD and NIPS membranes.** The blue, red and black arrows point to the characteristic peaks of the α, β and γ phase PVDF, respectively. NIPS membranes all show intensive peaks of α phase, together with peaks from β and γ phase. But for the CCD membranes, all peaks of α phase disappeared.

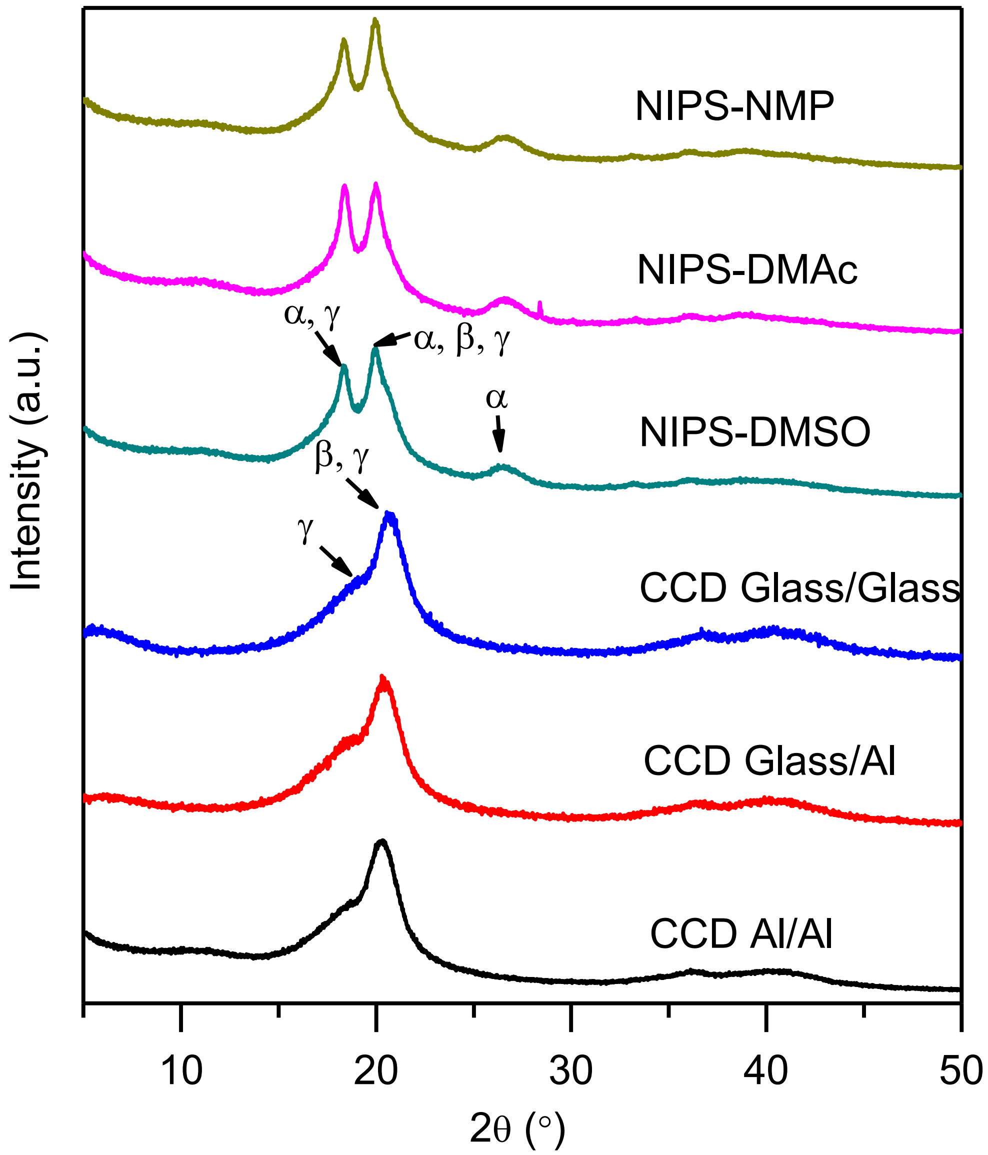


**Supplementary Figure 9. XRD patterns of the CCD and NIPS membranes.**

a

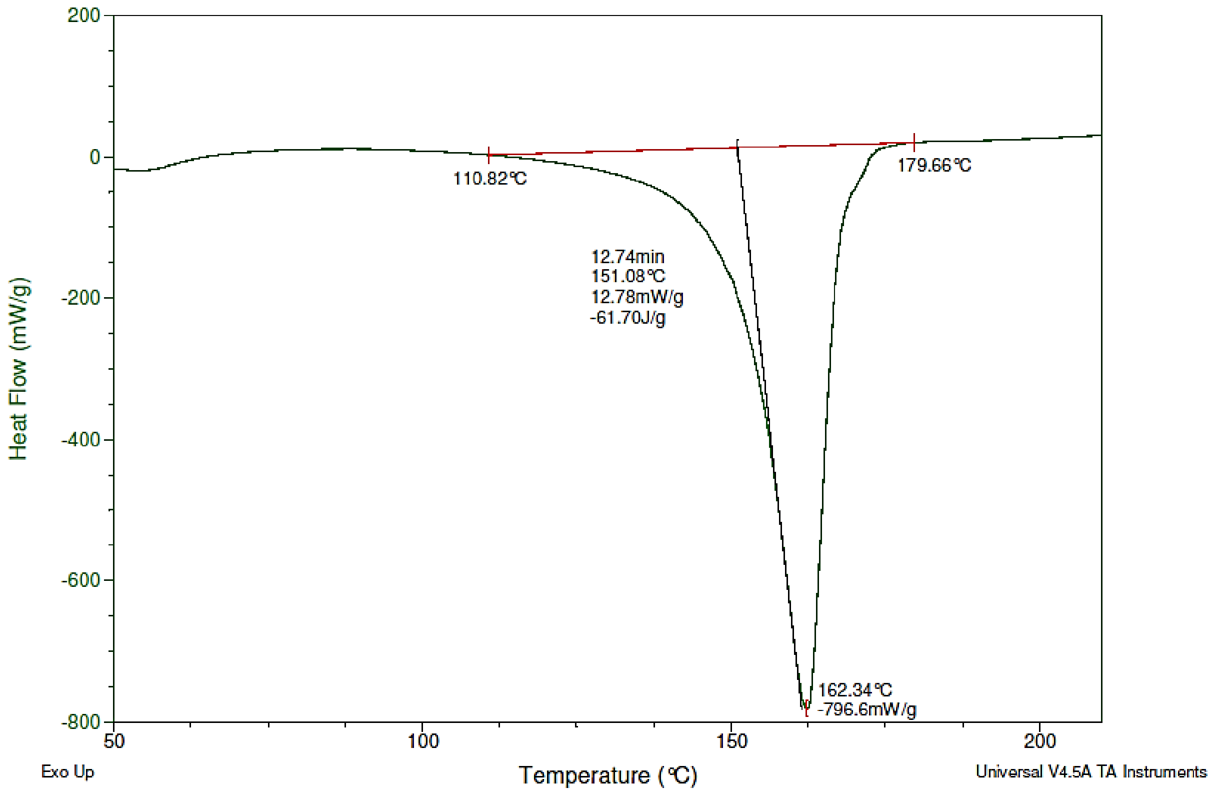
200
0
-200
-400
-600
-800
Heat Flow (mW/g)
110.82°C
179.66°C
12.74min
151.08°C
12.78mW/g
-61.70J/g
162.34°C
-796.6mW/g
50
100
150
200
Exo Up
Temperature (°C)
Universal V4.5A TA Instruments

b

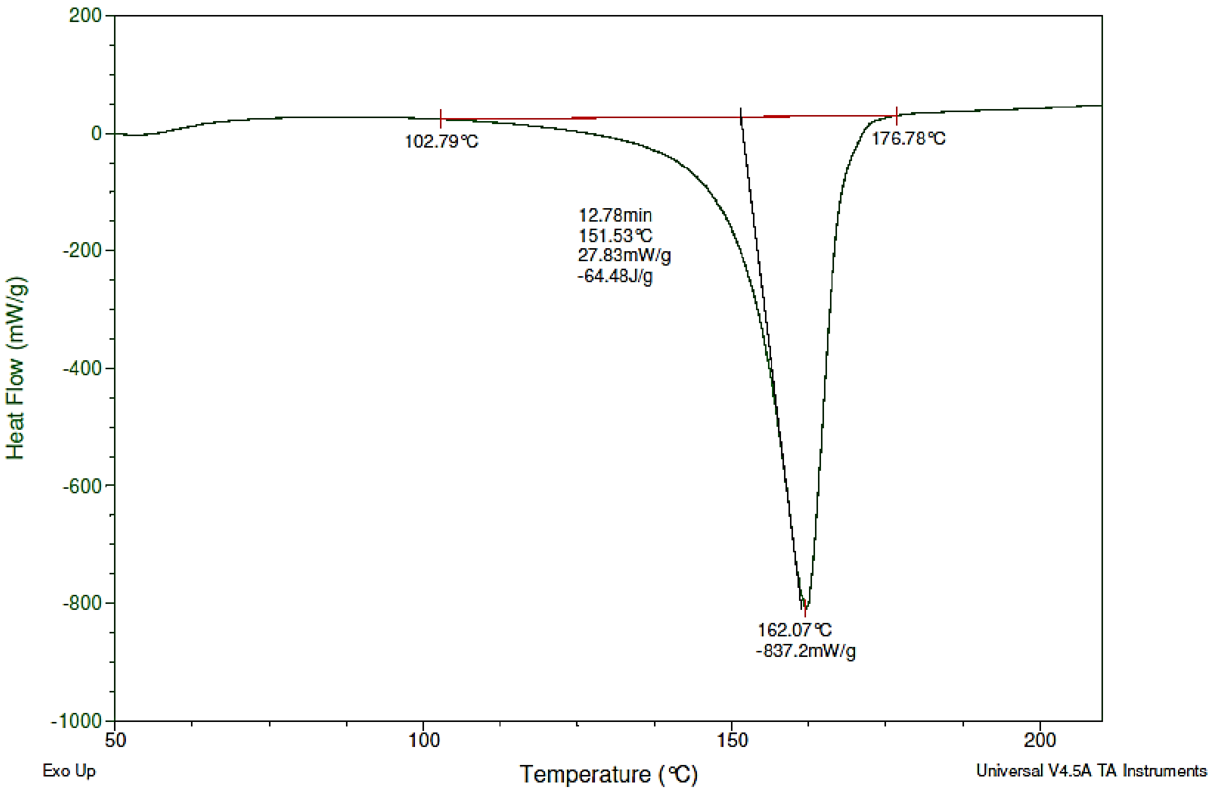
200
0
-200
-400
-600
-800
-1000
Heat Flow (mW/g)
102.79°C
176.78°C
12.78min
151.53°C
27.83mW/g
-64.48J/g
162.07°C
-837.2mW/g
50
100
150
200
Exo Up
Temperature (°C)
Universal V4.5A TA Instruments

c

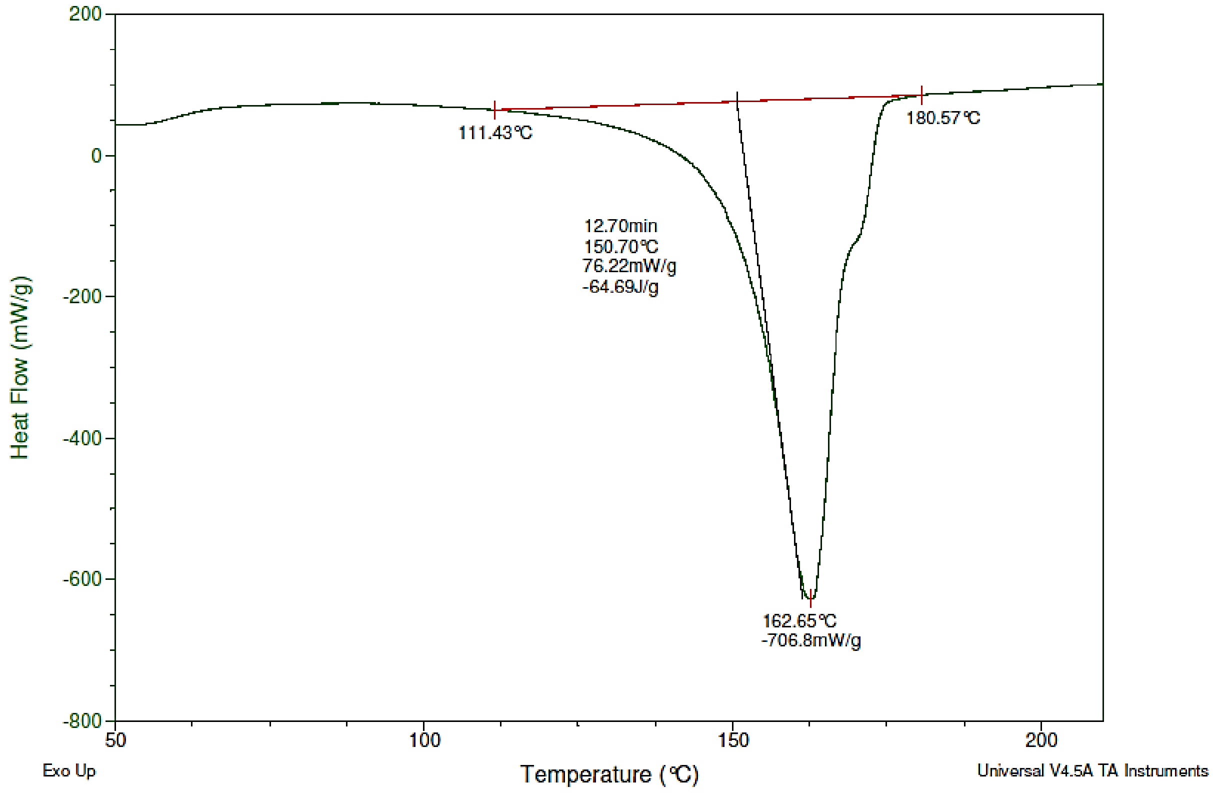


d

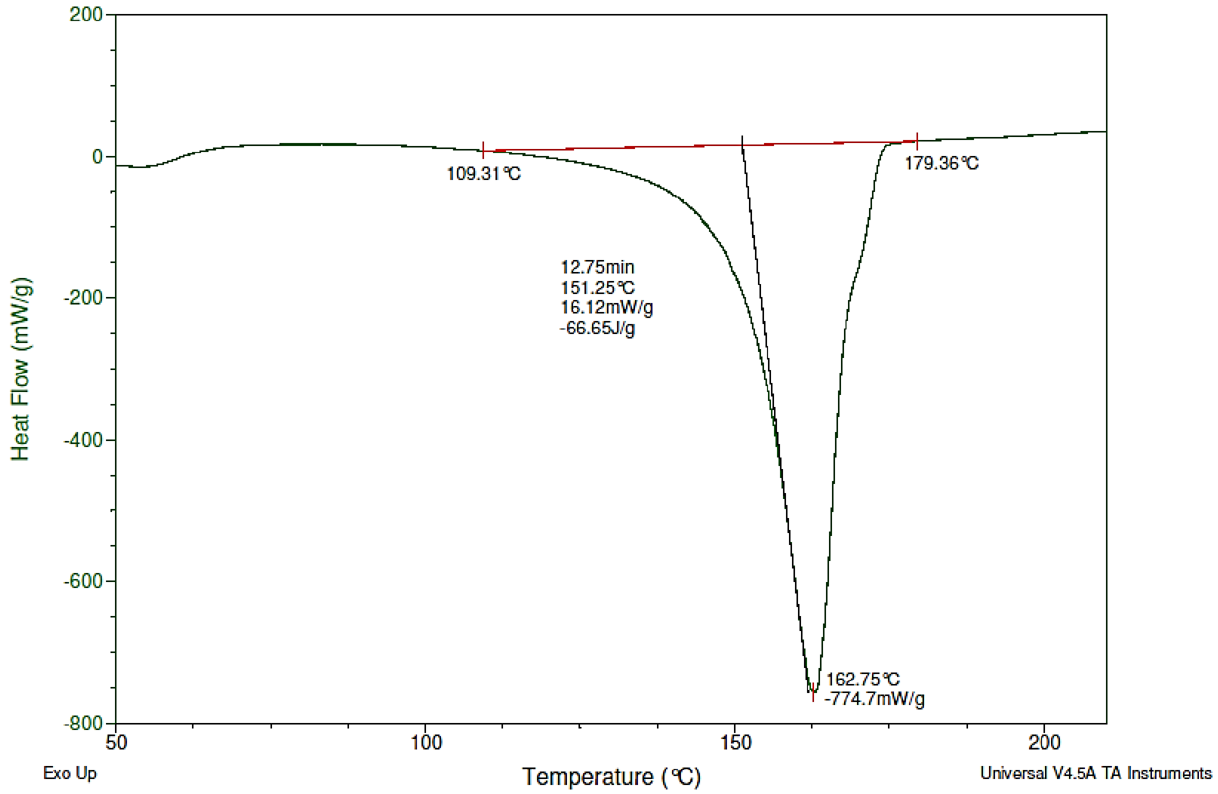


**Supplementary Figure 10 DSC results of the CCD membranes.** (**a**) Al/Al 1.0 mm, crystallinity 59%; (**b**) Al/Al 0.5 mm, crystallinity 62%; (**c**) Glass/Al 1.0 mm, crystallinity 62%; (**d**) Glass/Glass 1.0 mm, crystallinity 64%.

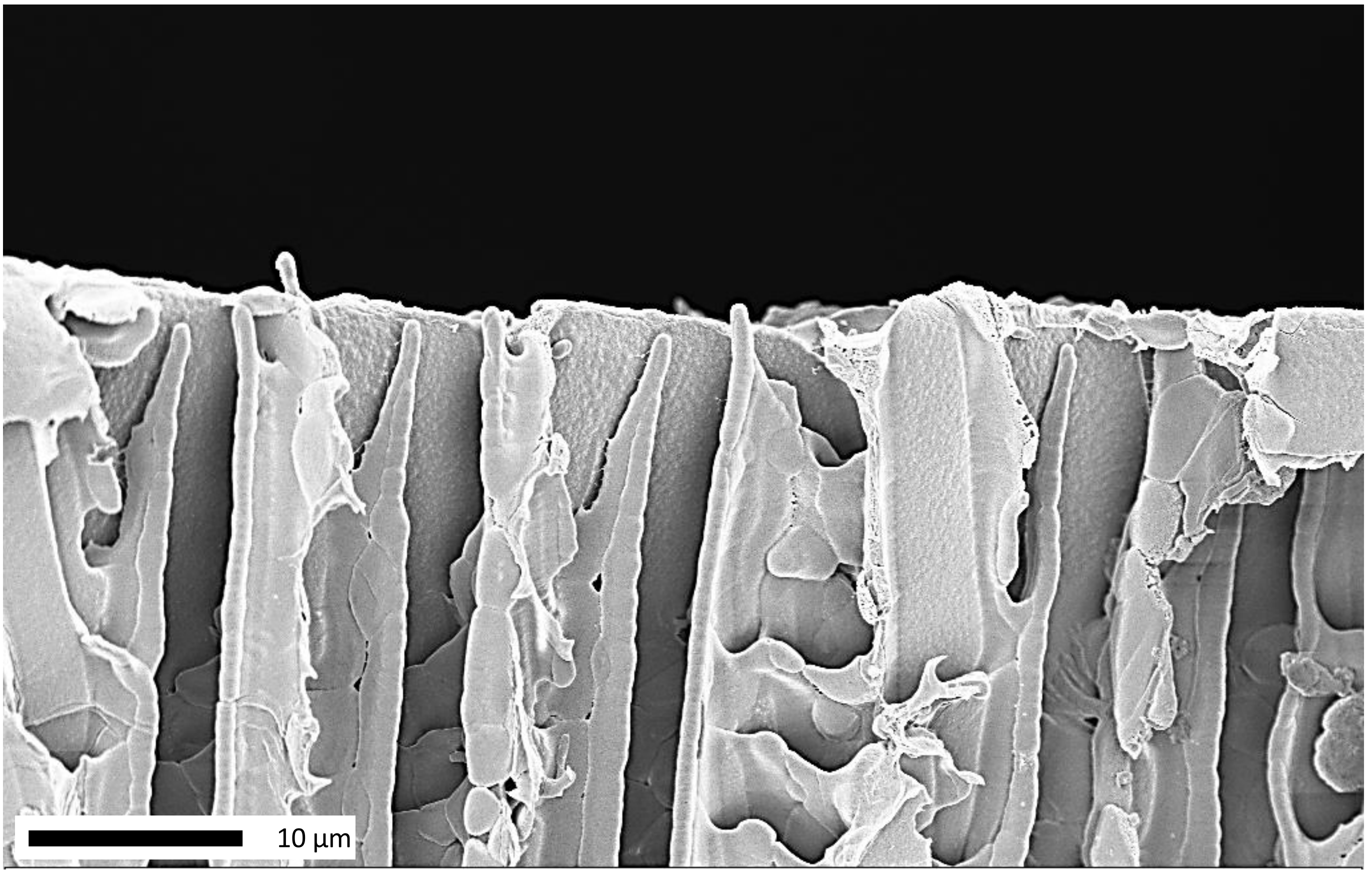


**Supplementary Figure 11. SEM image of the CCD Al/Al 1.0 mm PVDF membrane.** The image shows the cross section view close to the back side, which clearly depicted tightly connected PVDF grains and the grain boundary.

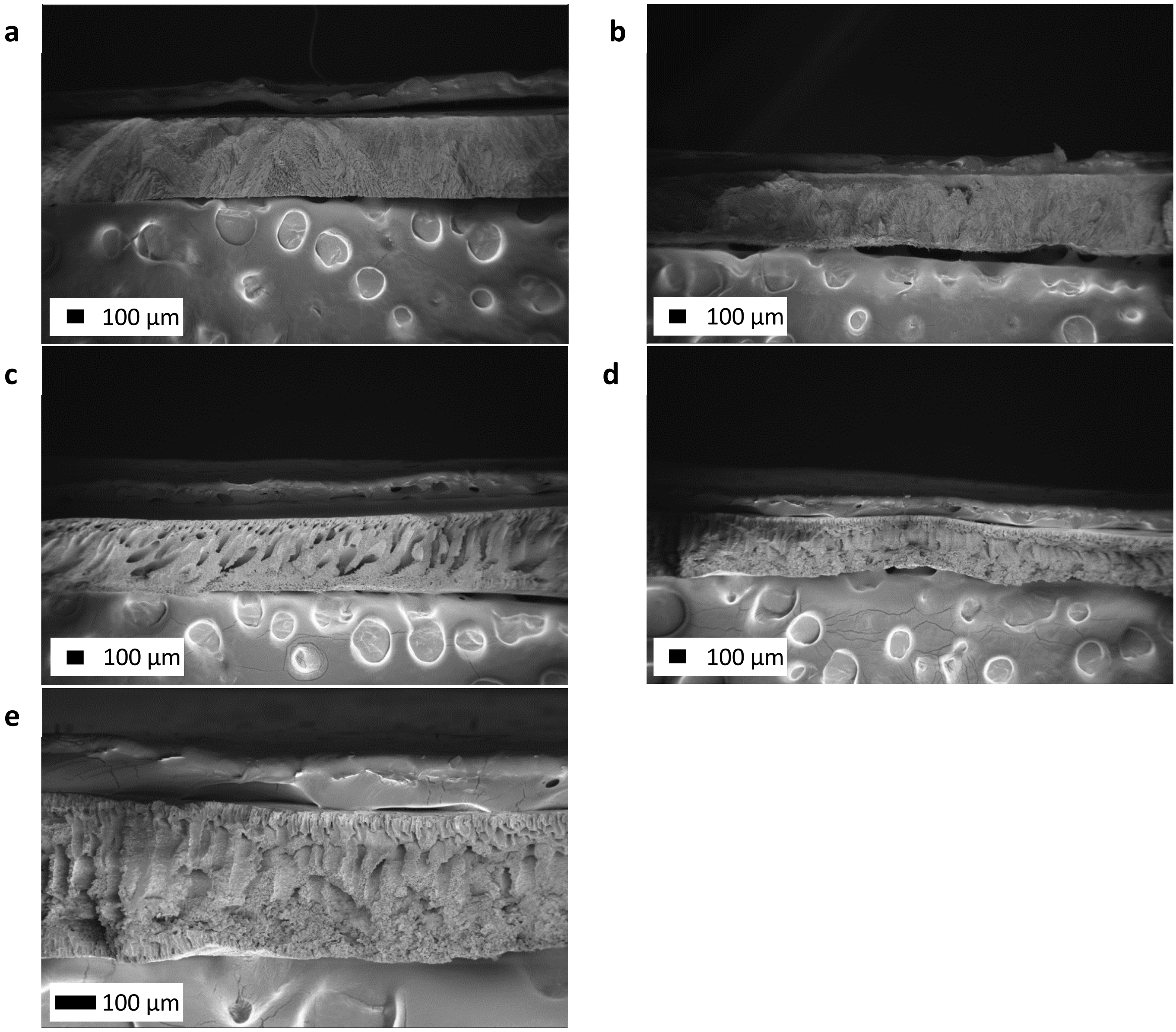


**Supplementary Figure 12. CCD Al/Al 1.0 mm PVDF membrane and NIPS DMSO 1.0 mm PVDF before and after high-pressure gas-liquid displacement measurements that reached 34.5 bar.** (**a**) Untested CCD membrane, thickness 460±5 µm;(**b**) tested CCD membrane, thickness 435±15 µm; (**c**) untested NIPS membrane, thickness 440±20 µm; and (**d, e**) tested NIPS membrane, thickness 330±30 µm. (**a-d**) are under same magnification, and (**e**) is enlarged view of (**d**). The tested NIPS membrane was obviously compressed and the lower part of the finger-like macrovoids collapsed.

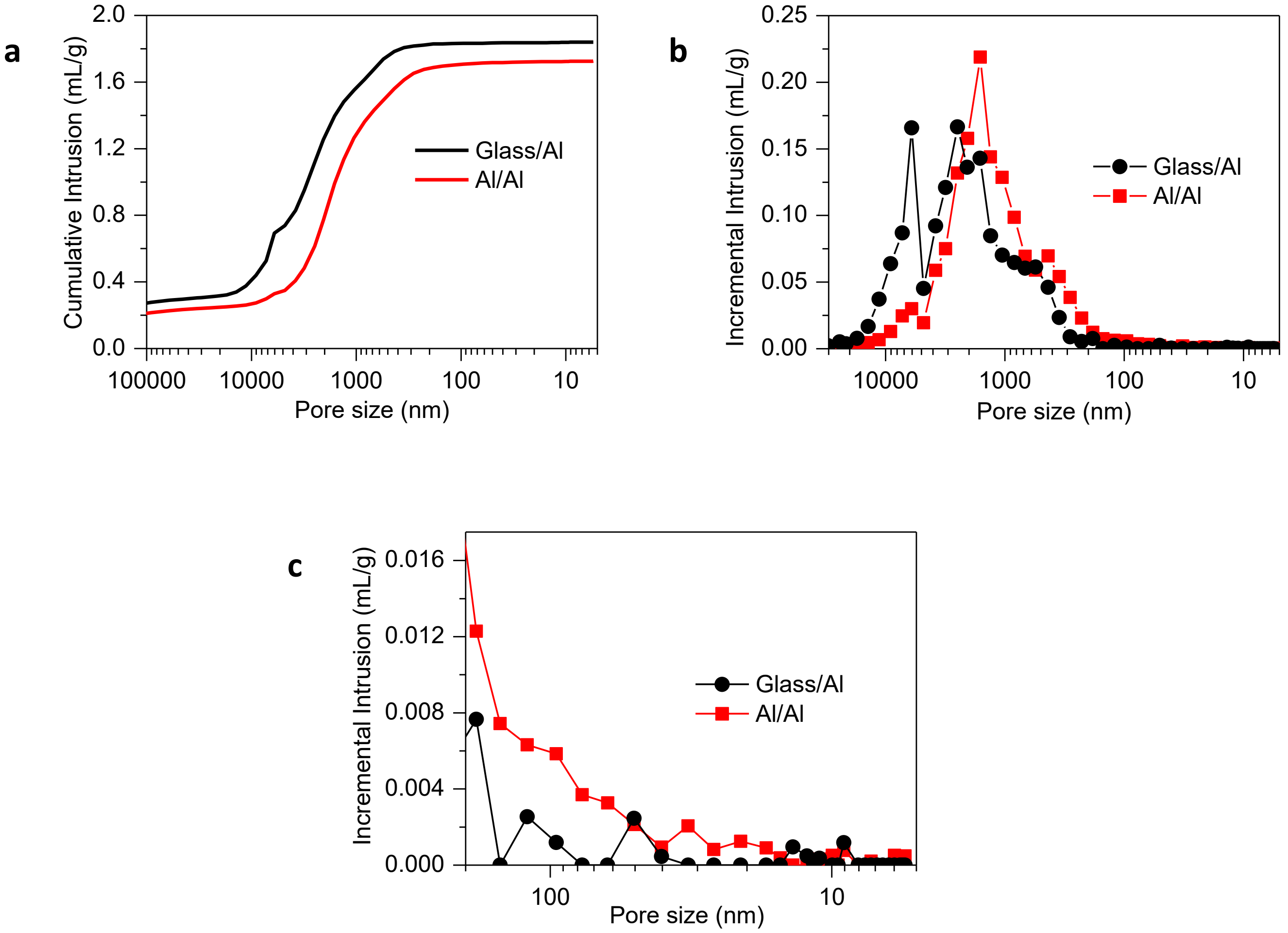


**Supplementary Figure 13. Mercury intrusion porosimetry results of CCD Glass/Al 1.0 mm membrane and Al/Al 1.0 mm membrane.** (**a**) Cumulative intrusion volume vs. pore size; (**b**) incremental intrusion volume vs. pore size; (**c**) incremental intrusion volume vs. pore size within small pore size range;

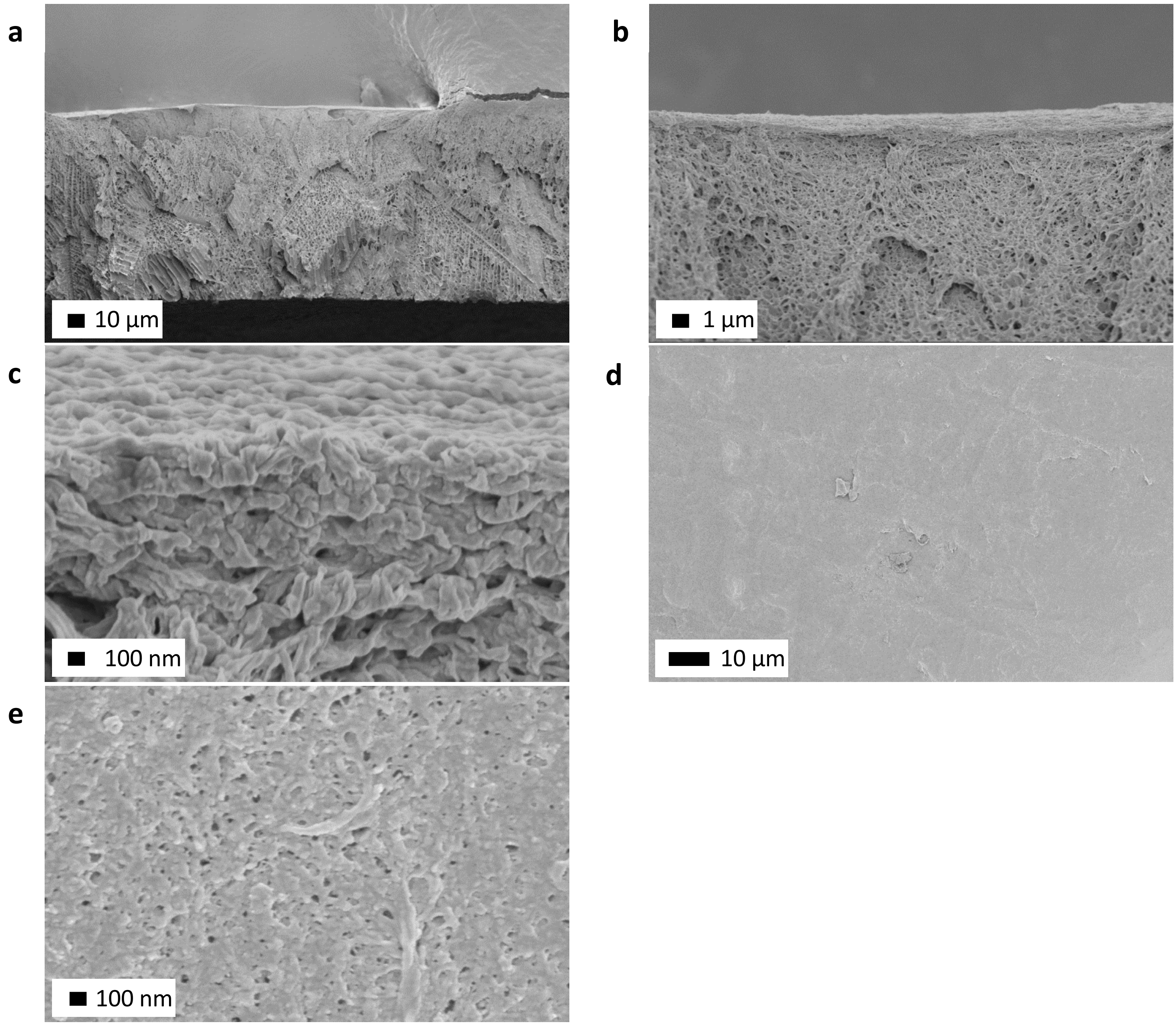


**Supplementary Figure 14. SEM images of a CCD Al/Al 0.3 mm PVDF membrane after the abrasion test for 2 weeks.** (**a**) The cross-sectional overview; (**b**) a cross-sectional view close to the separation layer; (**c**) high magnification image of the top separation layer; (**d**) overview of the membrane surface; and (**e**) high magnification image of the surface.

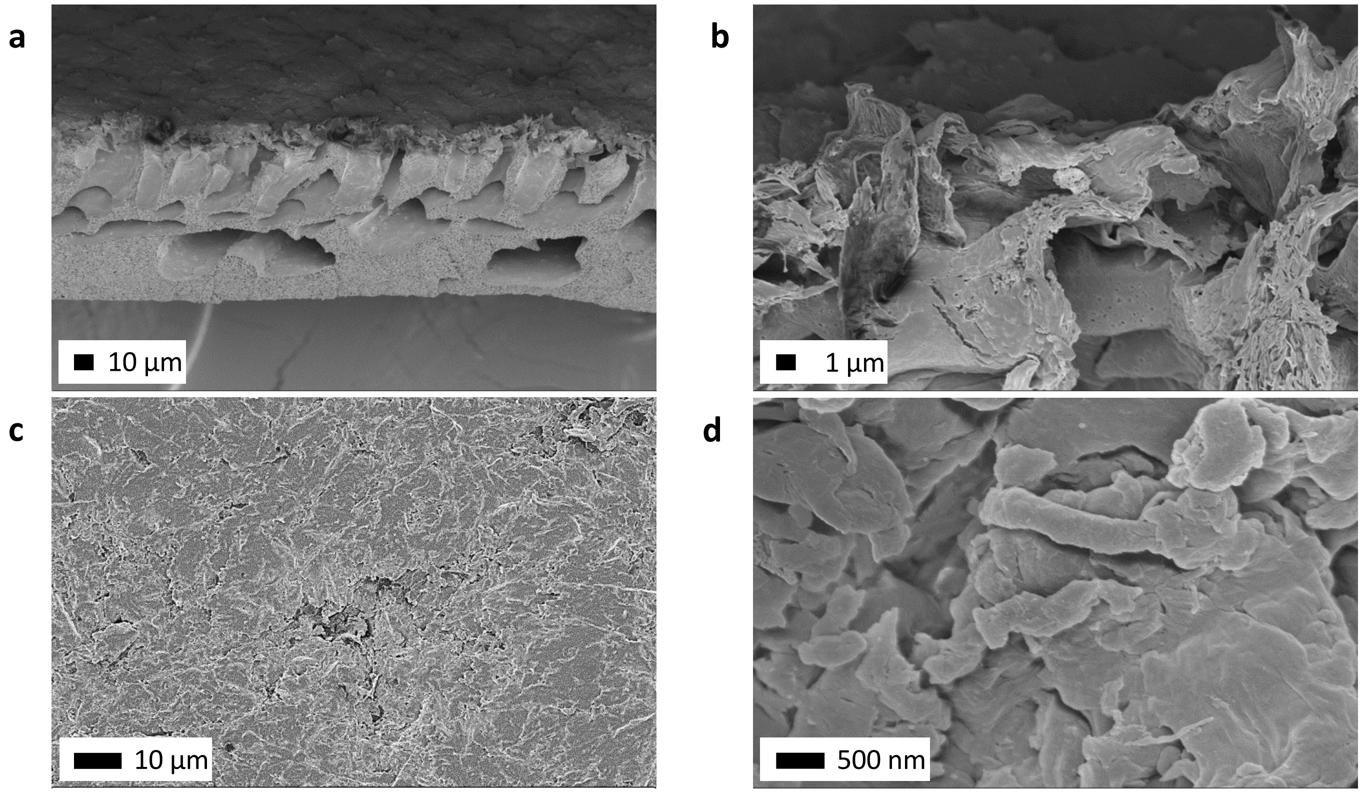


**Supplementary Figure 15. SEM images of a NIPS DMSO 0.3 mm PVDF membrane after the abrasion test for 2 weeks.** (**a**) The cross-sectional overview; (**b**) a cross-sectional view close to the separation layer; (**c**) overview of the membrane surface; and (**d**) a closer view on the surface.

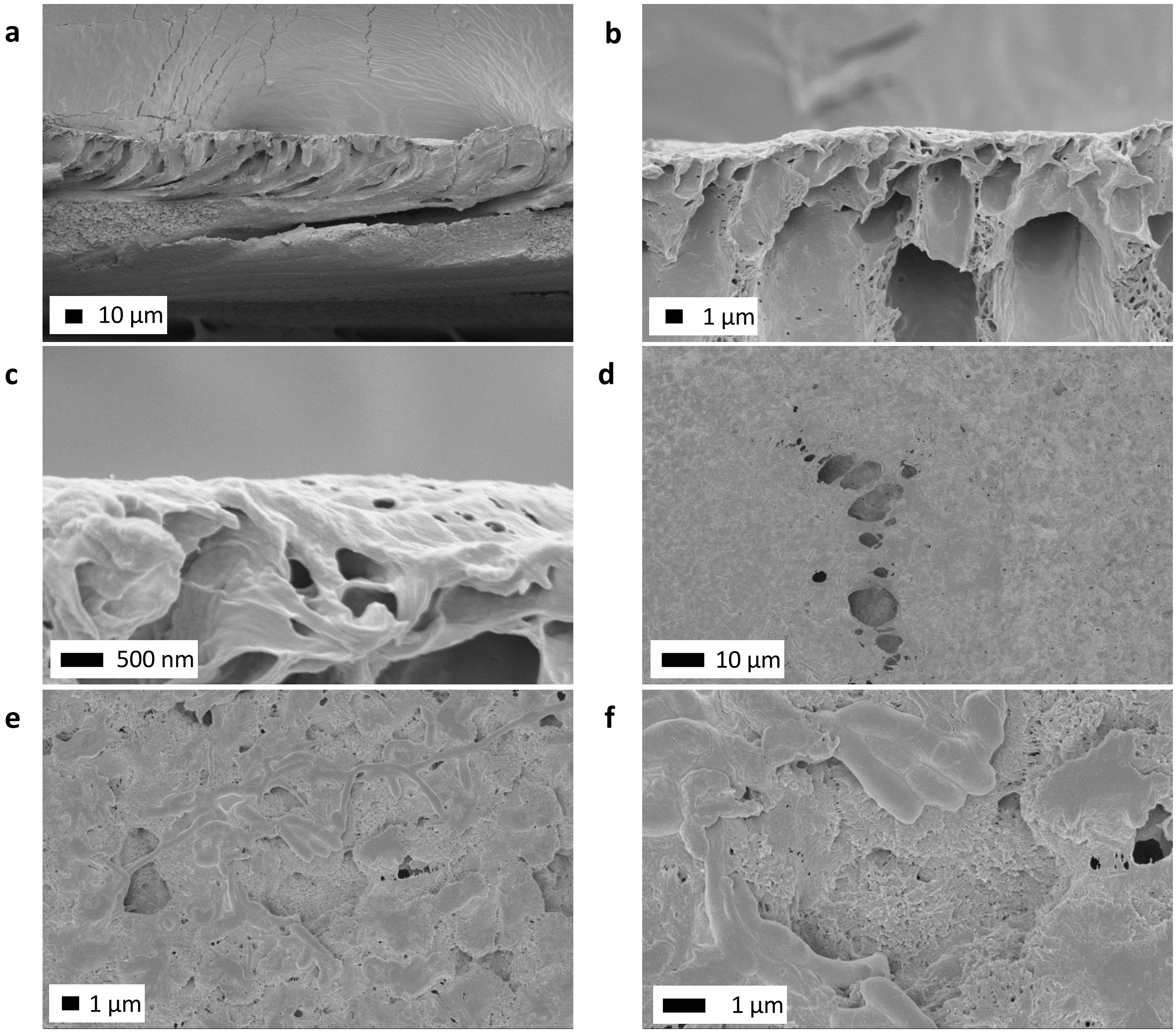


**Supplementary Figure 16. SEM images of a NIPS NMP 0.3 mm PVDF membrane after the abrasion test for 2 weeks.** (**a**) The cross-sectional overview; (**b**) a cross-sectional view close to the separation layer; (**c**) high magnification image of the top separation layer; (**d**) overview of the membrane surface; (**e**) a closer view on the surface; and (**f**) high magnification image of the surface.

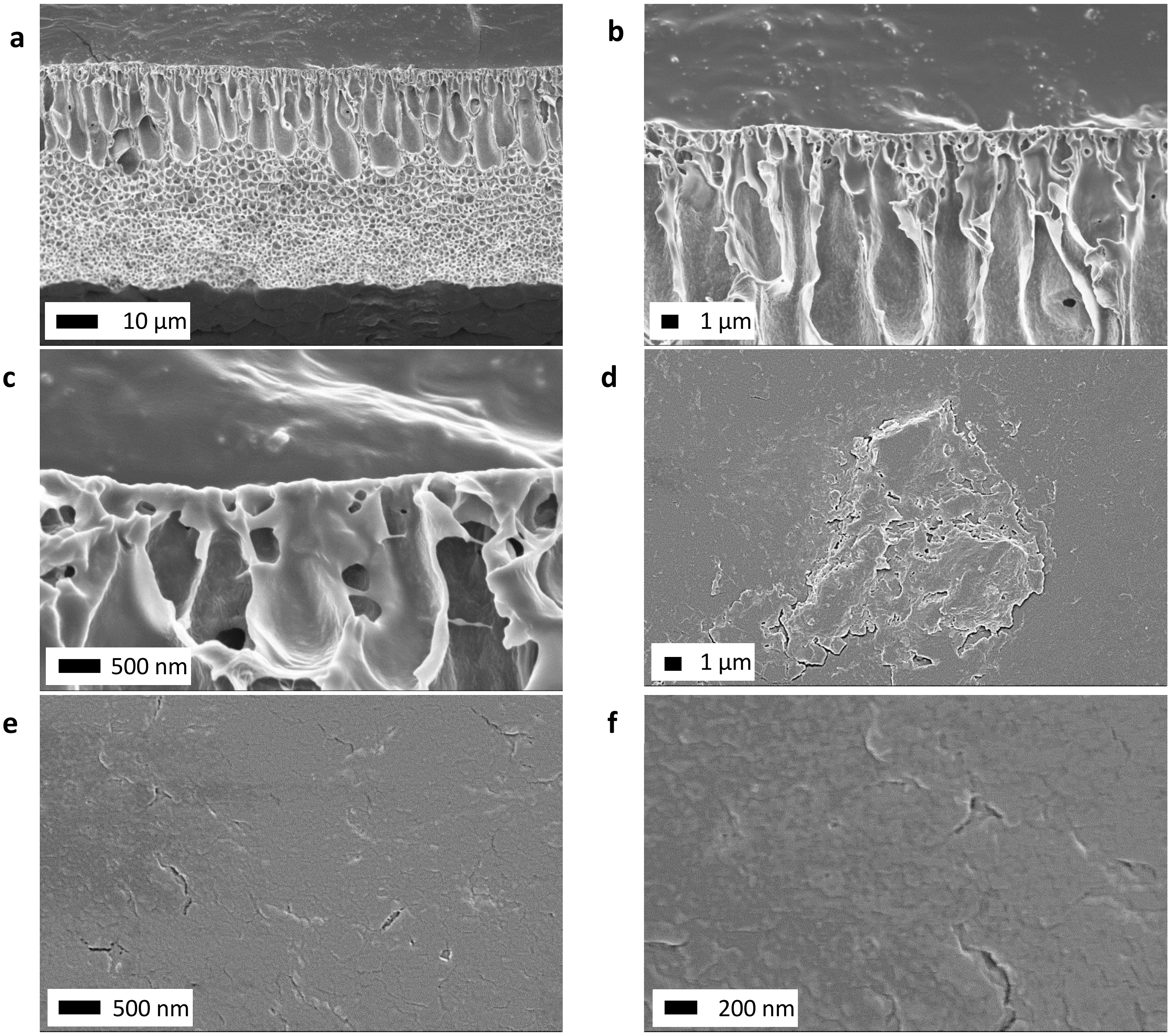


**Supplementary Figure 17. SEM images of a NIPS DMAc 0.3 mm PVDF membrane after the abrasion test for 2 weeks.** (**a**) The cross-sectional overview; (**b**) a cross-sectional view close to the separation layer; (**c**) high magnification image of the top separation layer; (**d**) overview of the membrane surface; (**e**) a closer view on the surface; and **f**) high magnification image of the surface.

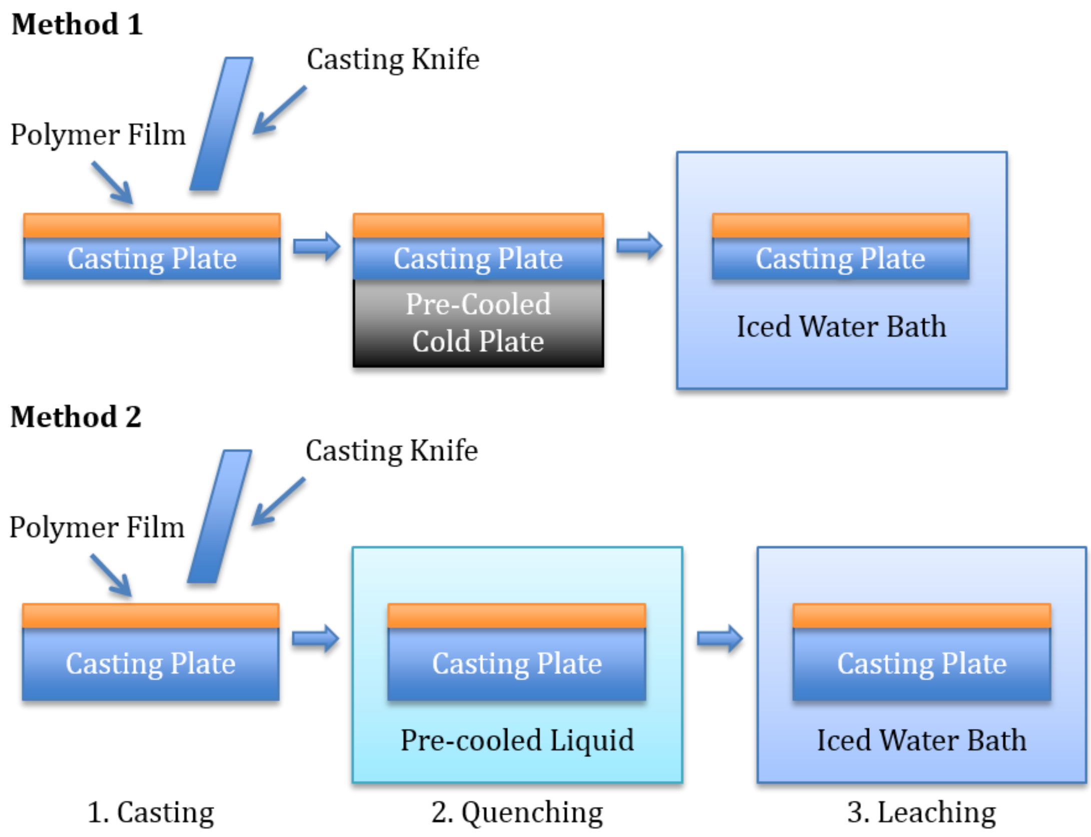


**Supplementary Figure 18 Schematic of the membrane preparation processes**

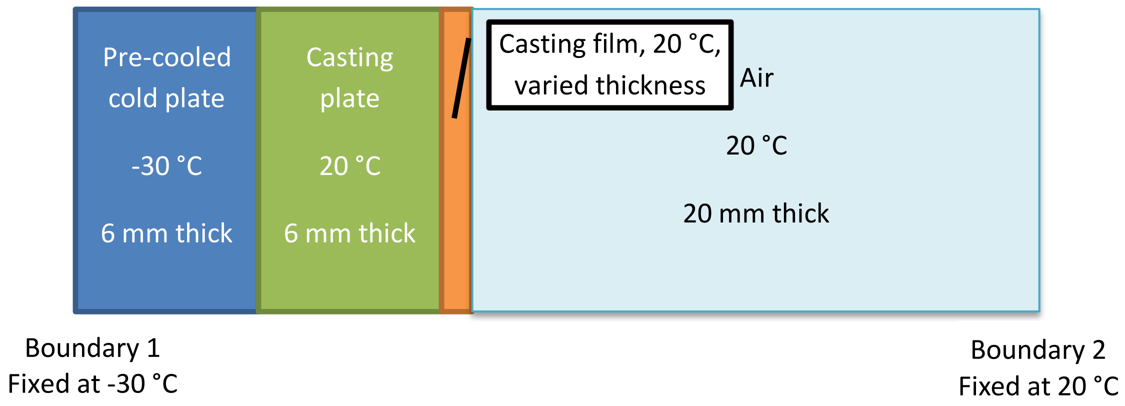


**Supplementary Figure 19. The setting of initial conditions for the calculation of thermal conduction under the circumstance of unidirectional cooling with a pre-cooled cold plate.**

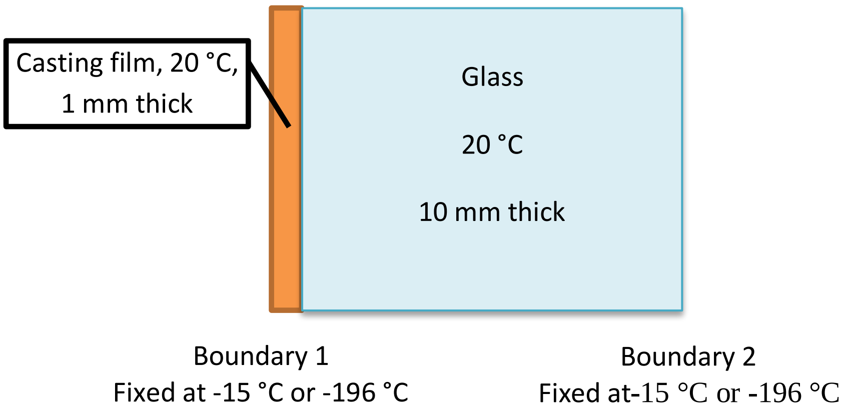


**Supplementary Figure 20. The setting of initial conditions for the calculation of thermal conduction under the circumstance of unidirectional cooling with a pre-cooled liquid.**

**Supplementary Table 1. Comparison of cross-flow BSA fouling test results for pure PVDF membranes made by NIPS, TIPS and CCD methods.**

| Ref | Membrane preparation method | Experiment conditions | Results |
|---|---|---|---|
| 1 | TIPS | Pure PVDF, 50 nm pore size, BSA 1g/L | BSA flux decreased from ~80 LMH to ~27 LMH (33.7%) in 2h. |
| 2 | NIPS | Bare PVDF, 20 nm pore size, BSA 1g/L | BSA flux decreased from ~100 to ~30 LMH at 0.05 $m^3$ permeated water per $m^2$ membrane area. |
| 3 | NIPS | Pure PVDF, BSA 1g/L, unknown pore size | Water permeation flux decreased from ~40 to 10 LMH when the feed was switched from pure water to BSA solution. |
| 4 | NIPS | Pure PVDF, unknown pore size, MWCO>>162 kDa, BSA 1g/L | BSA flux decreased to <10% in 2.5 h. The real flux was not given. |
| This work | CCD Al/Al | Pure PVDF, 45 nm pore size, BSA 1g/L | BSA flux decreased from 300 to 190 LMH in 1 h, 180 LMH in 2 h, and a stable flux of 100 LMH after 24 h. |

**Supplementary Table 2. Tensile test results of CCD membranes**

| **Sample name** | **Al/Al 1.0 mm** | **Glass/Al 1.0 mm** | **Glass/Glass 1.0 mm** |
|---|---|---|---|
| Maximum Load (N) | 12.0 ± 3.0 | 9.0 ± 2.8 | 8.0 ± 0.8 |
| Elongation at Maximum Load (%) | 47.8 ± 5.9 | 26.3 ±4.9 | 16.4 ± 6.8 |
| Tensile Stress (Mpa) | 2.4 ± 0.6 | 1.9 ± 0.6 | 1.9 ± 0.2 |
| Young's Modulus (Mpa) | 68.1 ± 12.8 | 75.2 ± 13.1 | 66.5 ± 9.2 |
| Maximum water speed* (m/s) | 23.6 | 20.1 | 18.8 |

* calculated based on a flat-sheet membrane of dimensions 1 × 2 $m^2$ (width × length), water flows along the length direction.

**Supplementary Table 3. Comparison between the NIPS and CCD methods for flat-sheet PVDF membrane production**

| | NIPS | CCD |
|---|---|---|
| **Preparation Method** | The casting film of polymer solution together with the support is immersed in a coagulation bath composed of a non-solvent for the polymer. The polymer solution is then transformed from a liquid to a solid state due to the exchange of the solvent in the polymer solution with the non-solvent from the coagulation bath. | The casting film of polymer solution is unidirectionally cooled from one side to a certain temperature far below the freezing point of the solvent. As a result, the solvent starts nucleation and crystallization, and the polymer precipitates to form the final membrane structure. Then the solvent is leached out by iced water. |
| **Typical Structure** | Asymmetric structure with a dense skin layer supported by finger-like voids and sponge-like layer | A thin separation layer of numerous tortuous pores supported by gradually changed, fully opened, interconnected and self-organized micro-channels |
| **Possible influencing factors during operation with a fixed composition of polymer solution** | - Composition of the coagulation bath medium;<br>- Original temperature of the polymer solution;<br>- Temperature of the casting plate;<br>- Coagulation bath temperature;<br>- Evaporation time;<br>- Ambient temperature and humidity;<br>- Casting thickness;<br>- Viscosity of the polymer solution and coagulation bath;<br>- Density of the polymer solution and coagulation bath;<br>- Other hydrodynamic factors (that influence interfacial instability): hydraulic pressure, the manner of immersing casting film into coagulation bath | - Cooling temperature;<br>- Cooling rate<br>- Casting thickness |
| **Permeation flux** | Very low | high |
| **Pore size distribution** | broad | sharp |
| **Transport resistance from support** | high | Very low |
| **Anti-fouling property** | poor | good |
| **Anti-wearing property** | poor | good |
| **Pore structure stability** | poor | excellent |

**Supplementary Table 4. Casting conditions for the preparation of flat sheet membranes**

| Sample | | solvent | Casting Thickness | Casting Plate | Cold plate/Cooling Condition |
|---|---|---|---|---|---|
| **CCD pure PVDF membranes** | **Glass/Glass** | DMSO | 1mm | 6 mm Glass | 6 mm Glass plate at -30 °C |
| | **Glass/Al** | DMSO | 1mm | 6 mm Al | 6 mm Al plate at -30 °C |
| | **Al/Al 1.0 mm** | DMSO | 1mm | 6 mm Al | 6 mm Al plate at -30 °C |
| | **Al/Al 0.5 mm** | DMSO | 0.5mm | 6 mm Al | 6 mm Al plate at -30 °C |
| | **Al/Al 0.3 mm** | DMSO | 0.3mm | 6 mm Al | 6 mm Al plate at -30 °C |
| | **Al/Al 0.1 mm** | DMSO | 0.1mm | 6 mm Al | 6 mm Al plate at -30 °C |
| | **Hexane** | DMSO | 1mm | 10 mm Glass | Hexane bath at -15 °C |
| | **Liquid $N_2$** | DMSO | 1mm | 10 mm Glass | Liquid nitrogen (-196 °C) |
| | **Al/Al NMP** | NMP | 1 mm | 6 mm Al | 6 mm Al plate at -30 °C |
| | **Al/Al DMAc** | DMAc | 1 mm | 6 mm Al | 6 mm Al plate at -30 °C |
| **CCD modified PVDF-PEG membranes** | **Glass/Al** | DMSO | 1mm | 6 mm Al | 6 mm Al plate at -30 °C |
| | **Al/Al 0.3 mm** | DMSO | 0.3mm | 6 mm Al | 6 mm Al plate at -30 °C |
| **NIPS pure PVDF membranes** | **DMSO 1.0 mm** | DMSO | 1.0mm | 6 mm Glass | N/A |
| | **DMSO 0.5 mm** | DMSO | 0.5mm | 6 mm Glass | N/A |
| | **DMSO 0.3 mm** | DMSO | 0.3mm | 6 mm Glass | N/A |
| | **DMAc 0.3 mm** | DMAc | 0.3mm | 6 mm Glass | N/A |
| | **NMP 0.3 mm** | NMP | 0.3mm | 6 mm Glass | N/A |

**Supplementary Note 1**

The NMP Al/Al membrane shows a thick dense separation layer and a sponge-like structure in the supporting layer, and no micro-channels were formed. This thick and dense separation layer showed a very low pure water flux of 6.5 LMH $bar^{-1}$, and no pores larger than 18.6 nm were detected with gas-liquid displacement porosimetry.

The DMAc Al/Al membrane also shows a homogeneous but porous supporting layer and a denser top layer. But this membrane broke apart when the crystallisation of DMAc is finished and only debris were obtained, which might due to the damaging shape of DMAc crystal grains that cuts the membrane. The SEM images show some deep cracks formed at the back side of the membrane. Permeation characteristics such as pore size and pure water flux were therefore not obtained for this membrane.

**Supplementary Note 2**

Supplementary Fig. 3 shows typical pore size distributions of CCD PVDF membranes and NIPS PVDF membranes. All measurements used same pressure steps to ensure fair comparisons. Supplementary Fig. 3**a** shows a CCD Glass/Glass 1 mm sample, which has a sharp peak at 308 nm with a percent flow of 97.2%; Supplementary Fig. 3**b** shows a CCD Glass/Al 1 mm sample, which has a sharp peak at 103 nm with a percent flow of 98.3%; and Supplementary Fig. 3**c** shows a CCD Al/Al 1 mm sample that has a peak at 40 nm with a percent flow of 72.0%. On the other hand, the membranes prepared by the NIPS method showed much broaden pore size distributions. The NIPS DMSO 1mm sample showed a maximum percent flow of 18.9% at 38 nm (Supplementary Fig. 3**d**), the NIPS DMSO 0.3 mm sample showed a maximum percent flow of 15.5% at 56 nm (Supplementary Fig. 3**e**), and the NIPS NMP 0.3 mm sample showed a maximum percent flow of only 8.4% at 57 nm (Supplementary Fig. 3**f**). The NIPS DMAc samples could not be measured, either due to the extremely low porosity, or because the pores are smaller than the testing limit of the equipment (18.6 nm).

**Supplementary Note 3**

In Supplementary Table 2, it is interesting to find that with a faster cooling rate used during the membrane fabrication process, the membrane shows better mechanical properties: higher fracture load, longer elongation and higher tensile stress. It is reasonable to attribute this trend to the microstructural change in the membrane due to the different cooling rates: with a faster cooling rate, the CCD membrane has smaller micro-channels, and the number of the micro-channel would be larger (this assumption agrees with the proposed membrane formation mechanism and is confirmed by SEM images). With smaller but more micro-channels, the stress would be better distributed in the membrane and the energy would be easier to be dissipated by deformation, and fatal damages would be less likely to happen.

**Supplementary Note 4**

The mercury intrusion results of the Al/Al and Glass/Al membranes show typical cumulative intrusion volume-pore size curves (Supplementary Fig. 13**a**) similar to those rigid pore structures such as in ceramic membranes, with an overall porosity of about 75-76 % and a broaden pore size distribution from around 20 µm to less than 0.1 µm. The gradually increased intrusion volume reflects the gradual change in the pore size from the backside to the top separation layer in the CCD membranes. As expected, the incremental intrusion data (Supplementary Fig. 13**b**) of the Al/Al membrane reveals a smaller pore size (11 µm) than the Glass/Al membrane (17 µm) at which intrusion starts, which correspond to the openings of the micro-channels on the backside. The average pore size of the Al/Al membrane is also smaller than the Glass/Al membrane. Closer observation of the incremental intrusion results (Supplementary Fig. 13**c**) shows that the Al/Al membrane has higher pore volume at the pore size range of less than 100 nm than the Glass/Al membrane. These results agree very well with SEM images and gas-liquid displacement porosimetry results, and also agree with the prediction of membrane structure based on the cooling rate.

**Supplementary Note 5**

With the experimental method employed for the abrasion tests, it is known that the most severe damages occur at the centre part of the membrane [5], therefore all SEM images given here were taken from the centre of the membranes for fair comparison

For the **CCD Al/Al 0.3 mm** membrane, it essentially kept the original pore structure in the separation layer and the whole membrane structure after the abrasion test. Although some extent of wearing can be found on the membrane surface, where debris were observed (Supplementary Fig. 14**d**), the pore size on the surface and in the separation layer were not affected (Supplementary Fig. 14**c&e**). And in the Supplementary Fig. 14**c**, it can be seen that the thickness of the separation layer basically didn't change compared with the untested same type of membrane shown in Fig. 2**c**, meaning that the wearing of the membrane under such accelerated test was very slight.

For the **NIPS DMSO 0.3 mm** membrane, the top separation layer was completely destroyed after the abrasion test. From the SEM images, it can be seen that there were only debris remained at the top layer and the separation layer was gone. And for the **NIPS NMP 0.3 mm** membrane, the extent of wearing is less than the DMSO sample and the top layer still remains. But the top layer has been largely deformed and the pore structure has been completely altered. Big holes appear on the membrane surface and the surface microstructure has become very rough with apparent worn parts. The **NIPS DMAc 0.3 mm** membrane was the least damaged sample among the NIPS samples after the abrasion test. The top layer remained almost unchanged after the test, but some debris can be seen on the surface. However, in high magnification SEM images, it is clear that intensive cracks start to appear on the membrane surface after the test, which would change the pore size and ruin the selectivity of the membrane.

## Supplementary references


1. Rajabzadeh S, Ogawa D, Ohmukai Y, Zhou Z, Ishigami T, Matsuyama H. Preparation of a PVDF hollow fiber blend membrane via thermally induced phase separation (TIPS) method using new synthesized zwitterionic copolymer. *Desalin Water Treat,* **54**, 2911-2919 (2015).

2. Nishigochi S*, et al.* Improvement of Antifouling Properties of Polyvinylidene Fluoride Hollow Fiber Membranes by Simple Dip Coating of Phosphorylcholine Copolymer via Hydrophobic Interactions. *Industrial & Engineering Chemistry Research,* **53**, 2491-2497 (2014).

3. Sui Y, Gao XL, Wang ZN, Gao CJ. Antifouling and antibacterial improvement of surface-functionalized poly(vinylidene fluoride) membrane prepared via dihydroxyphenylalanine-initiated atom transfer radical graft polymerizations. *J Membr Sci,* **394**, 107-119 (2012).

4. Boributh S, Chanachai A, Jiraratananon R. Modification of PVDF membrane by chitosan solution for reducing protein fouling. *J Membr Sci,* **342**, 97-104 (2009).

5. Ji J, Zhou S, Lai CY, Wang B, Li K. PVDF/palygorskite composite ultrafiltration membranes with enhanced abrasion resistance and flux. *J Membr Sci,* **495**, 91-100 (2015).
